# A Metabolic Immune Checkpoint: Adenosine in Tumor Microenvironment

**DOI:** 10.3389/fimmu.2016.00109

**Published:** 2016-03-29

**Authors:** Akio Ohta

**Affiliations:** ^1^Center for Drug Discovery, Northeastern University, Boston, MA, USA

**Keywords:** adenosine, tumor microenvironment, A2A adenosine receptor, CD73, tissue hypoxia, immunosuppression, immune checkpoint, cancer immunotherapy

## Abstract

Within tumors, some areas are less oxygenated than others. Since their home ground is under chronic hypoxia, tumor cells adapt to this condition by activating aerobic glycolysis; however, this hypoxic environment is very harsh for incoming immune cells. Deprivation of oxygen limits availability of energy sources and induces accumulation of extracellular adenosine in tumors. Extracellular adenosine, upon binding with adenosine receptors on the surface of various immune cells, suppresses pro-inflammatory activities. In addition, signaling through adenosine receptors upregulates a number of anti-inflammatory molecules and immunoregulatory cells, leading to the establishment of a long-lasting immunosuppressive environment. Thus, due to hypoxia and adenosine, tumors can discourage antitumor immune responses no matter how the response was induced, whether it was spontaneous or artificially introduced with a therapeutic intention. Preclinical studies have shown the significance of adenosine in tumor survival strategy by demonstrating tumor regression after inactivation of adenosine receptors, inhibition of adenosine-producing enzymes, or reversal of tissue hypoxia. These promising results indicate a potential use of the inhibitors of the hypoxia–adenosine pathway for cancer immunotherapy.

## Immune Checkpoint

The discovery of tumor-associated antigens and tumor-reactive immune cells led to a promise of a new cancer therapy based on tumor antigen-specific immune responses. Preventing successful immunotherapy of cancer is the immunosuppressive nature of the tumor microenvironment (1–3). In animals expressing the same antigen in both tumors and normal tissue, the intensity of antigen-specific T cell response was diminished in tumors, while the same T cells were vigorously activated in normal tissue (4, 5). T cells infiltrated in tumor tissue are often capable of recognizing tumor-associated antigen, but they coexist with their target, tumor cells, without significant antitumor activity. However, when isolated from tumor tissue, those tumor-infiltrated T cells could kill the tumor cells efficiently *in vitro* (6). These studies indicate that tumors establish a stern environment for antitumor immune cells, cells that can be active effector cells otherwise.

Many of the immunosuppressive mechanisms in tumors are common to physiological immunoregulation in normal tissues. Such immunoregulation is very important in keeping the immune system under control in order to block a self-reactive immune response and to prevent an ongoing immune response from causing critical tissue damage. The lack of physiological immunoregulation often results in overwhelming immune activation that accompanies autoimmunity. For example, CTLA-4 is a physiological mechanism that negatively regulates T cell activity by blocking a costimulatory signal through CD28–B7 interaction. The lack of CTLA-4 causes non-specific T cell activation, and CTLA-4-deficient mice die in several weeks with massive lymphocytic tissue infiltration (7, 8). In humans, heterozygous mutation in the CTLA-4 gene is enough to cause immune dysregulation similar to homozygous ­CTLA-4-knockout mice (9). PD-1 also provides a T cell inhibitory signal upon interaction with its ligands, PD-L1 and PD-L2. Deficiency of PD-1 in mice is known to cause various types of autoimmune disorders depending on the genetic strains (10).

Besides cell surface transducers of immunosuppressive signal, e.g., CTLA-4 and PD-1, immunosuppression in the tumor microenvironment involves anti-inflammatory cytokines (IL-10, ­TGF-β), enzymes (indoleamine-2,3-dioxygenase), and professional immunoregulatory cells [regulatory T cells, myeloid-derived suppressor cells (MDSCs)] (1, 2). These immunosuppressive mechanisms play an important role in controlling immune response in normal tissues, not tumor tissue specific. Since tumors take advantage of such physiological immunoregulatory mechanisms to protect their tissue from immune attack, these mechanisms intended to prevent inflammatory complication, now turn out to be major obstacles hampering spontaneous cancer regression and immunological cancer treatment. The identification of immunosuppressive mechanisms in tumors pointed out molecular targets to restore the antitumor immune response. Thus, these negative immunoregulatory mechanisms, so-called immune checkpoints, became a focus in drug discovery. The effort resulted in FDA approval of anti-CTLA-4 and anti-PD-1 antibodies for cancer treatment. This achievement finally convinced people that immunotherapy of cancer is realistic, and it further encouraged the development of inhibitors of other immune checkpoint molecules (10–12).

Extracellular adenosine has been known as an inhibitor of immune functions. While intracellular adenosine is involved in energy metabolism, nucleic acid metabolism, and the methionine cycle, extracellular adenosine plays an important role in intercellular signaling. Its signal is transmitted by G protein-coupled adenosine receptors on the cell surface, and it affects diverse physiological functions including neurological, cardiovascular, and immunological systems (13). Extracellular concentration of adenosine can increase in response to metabolic change. When cells are deprived of nutrients or oxygen, insufficient ATP biosynthesis tends to lower the ATP/adenosine ratio. To reduce ATP expenditure, cells may suspend energy-consuming activities such as cell proliferation, which requires biosynthesis of a large amount of cellular components (14, 15). Indeed, tissue hypoxia strongly represses proliferation of activated T cells (16). Interestingly, extracellular adenosine is known to accumulate under hypoxic conditions. Adenosine signaling may play a role in the improvement of energy status by promoting catabolism of stored metabolic energy. Correspondingly, extracellular adenosine increases energy expenditure through the induction of lipolysis (17). Tumors contain high levels of extracellular adenosine (18, 19), ­­suggesting that tumor cells may benefit from its immunosuppressive effect and catabolic energy production. The current review focuses on the ­pro-cancer roles of extracellular adenosine and discusses application of the inhibitors of this metabolic immune checkpoint to cancer immunotherapy.

## Adenosine Receptors and Suppression of Antitumor Immunity

Of the four known types of adenosine receptors, A2A adenosine receptor (A2AR) is the predominantly expressed subtype in most immune cells (13). Stimulation of A2AR generally provides an immunosuppressive signal that inhibits activities of T cells (proliferation, cytokine production, cytotoxicity), NK cells (cytotoxicity), NKT cells (cytokine production, CD40L upregulation), macrophages/dendritic cells (antigen presentation, cytokine production), and neutrophils (oxidative burst) (20, 21). Administration of A2AR agonist strongly inhibits induction of various inflammatory diseases (22, 23). Endogenous adenosine shows similar immunoregulatory activity via A2AR, and this mechanism is critical to the control of the immune response. Exaggeration of inflammatory tissue injury in A2AR-deficient mice indicated that no other immunoregulatory mechanism could compensate for the lack of the adenosine-dependent immunoregulation (20, 24–28).

In 2006, the presence of high levels of extracellular adenosine in tumors was found to play a significant role in the evasion of antitumor immune response (19). The study showed that A2AR-deficient mice could spontaneously regress the inoculated tumor, whereas no wild-type mice showed similar tumor regression. A2AR antagonists were also beneficial in tumor-bearing wild-type animals. Importantly, depletion of T cells and NK cells impaired the retardation of tumor growth by A2AR antagonists, suggesting improvement of antitumor cellular immune response (19, 29). Effector functions of T cells and NK cells are susceptible to A2AR stimulation. In addition, when activated in the presence of A2AR agonist, the effector function of T cells is persistently impaired even after removal of A2AR agonist (30, 31). This result suggests that the adenosine-rich environment in tumors may induce T cells that are anergic to the tumor cells. Consistent with this change, A2AR stimulation induces immunoregulatory molecules such as CTLA-4 and PD-1 on T cells (32–34). Antigen-presenting cells (APCs) are also targets of adenosine. A2AR agonists suppress IL-12 and induce IL-10 production from APCs, discouraging cellular immune response (35, 36). Conversely, myeloid-specific deletion of A2AR enhanced tumor infiltration of effector T cells and NK cells (37). These studies show A2AR-dependent suppression of antitumor T cell activity. However, a recent study reported an interesting finding that A2AR-deficient antitumor T cells might have a shorter half-life *in vivo* (38). A2AR-deficient T cells effectively elicit antitumor activities in the adenosine-rich tumor microenvironment, but if these cells were to disappear prematurely, they may not complete tumor regression and may actually result in tumor regrowth. Further studies with this issue may result in an improved ­treatment in which adenosine-resistant antitumor effector cells can offer persistent immune response.

Furthermore, adenosine recruits immunoregulatory activity of regulatory T (Treg) cells. FoxP3, a key transcriptional factor for the immunosuppressive activity of Treg cells, is inducible by A2AR stimulation (30). T cell activation in the presence of A2AR stimulation largely increases CD4^+^ FoxP3^+^ cells (33, 39–41). Interestingly, in addition to the numerical increase of Treg cells, A2AR stimulation augmented the immunoregulatory activity of Treg cells (33). Consistent with the increase in regulatory activity *in vitro*, Treg cell-dependent prevention of *in vivo* inflammatory disease induction could be enhanced by pretreating Treg cells with A2AR agonist before transfer (39). In contrast, transfer of A2AR-deficient Treg cells failed to save the tissue from inflammatory damage (42), suggesting that endogenous adenosine may be important in the modulation of Treg activity *in vivo*.

A2B adenosine receptor (A2BR) can also potentially mediate immunosuppressive activity of extracellular adenosine, but the mechanisms of A2BR-dependent immunosuppression are different from those of A2AR. A2BR-dependent immunoregulation is notable in myeloid cells. A2BR plays a predominant role in the adenosine-dependent differentiation of macrophages into M2-type (43–45). Those macrophages activated in the presence of A2BR stimulation express arginase, indoleamine-2,3-dioxygenase, and TGF-β, and have limited T cell stimulatory activity. In addition to the induction of such tolerogenic APCs, the adenosine–A2BR interaction results in the expansion of MDSCs (46). Indeed, treatment of tumor-bearing mice with A2BR agonist increased MDSC in tumors and accelerated tumor growth (47).

Immunoregulatory activity of A2BR is also involved in the suppression of antitumor immunity by adenosine present in the tumor microenvironment. Slower tumor growth was observed in A2BR-deficient mice and A2BR antagonist-treated wild-type mice (48, 49). Recruitment of T cell immunity was necessary to provoke antitumor effect of A2BR antagonist because the treatment was not effective in T cell-deficient animals (49). Diminished tumor growth by A2BR antagonist accompanied a greater number of T cell infiltration but reduced numbers of Treg cells and MDSC (47, 49, 50).

Thus, extracellular adenosine downregulates antitumor immune response in various ways. The mechanisms of immunosuppression involve not only direct effects on antitumor effector cells but also indirect effects on APCs and professional immunoregulatory cells such as Treg cells and MDSC. A2AR and/or A2BR mediate immunosuppressive effects of adenosine depending on cell types. Interestingly, A2AR and A2BR seem to mainly target lymphoid cells and myeloid cells, respectively. The impact of adenosine-mediated immunosuppression seems to be persistent rather than transient because the outcome of adenosine exposure can induce M2-type tumor-associated macrophages, Treg cells, MDSC, and “anergic” effector T cells. These facts support an idea that extracellular adenosine is a negative immune checkpoint molecule that plays a significant role in establishing an immunosuppressive tumor microenvironment. Therefore, it is reasonable to target the adenosine-dependent pathway in order to improve cancer therapy. The blockade of A2AR and A2BR using antagonists has demonstrated promise for a new therapeutic approach (Table 1).

**Table 1:** Representative A2AR/A2BR antagonists (majority of A2AR antagonists were developed for Parkinson’s disease; *under cancer clinical trial).

	A2AR	A2BR
Clinical	CPI-444*	CVT-6883
	PBF-509*	
	Istradefylline (KW-6002)	
	Preladenant (SCH420814)	
	Tozadenant (SYN115)	
	Vipadenant (BIIB014)	
	HTL-1071	
	ST1535	
Others	SCH412348	MRE2029F20
	SCH442416	MRS1754
	SCH58261	PSB603
	ZM241385	

## Cancer Immunotherapy by Targeting Adenosine-Producing Mechanism

To inactivate the adenosine-dependent immunosuppression, it would be beneficial to think about the metabolism of extracellular adenosine. The source of extracellular adenosine is believed to be ATP in the extracellular compartment. There are nucleotidases on the cell surface called CD39 and CD73. CD39 catalyzes hydrolysis of ATP/ADP to AMP, and CD73 converts AMP to adenosine. Very low extracellular adenosine levels in CD73-deficient mice suggest that degradation of ATP is a major source of extracellular adenosine (51, 52). Adenosine is metabolized to inosine by adenosine deaminase or converted to AMP by the function of adenosine kinase. Tissue hypoxia can induce accumulation of extracellular adenosine by increasing adenosine production and by decreasing adenosine removal. Hypoxia induces the mRNA levels and enzymatic activities of CD39 and CD73 leading to an increase in adenosine production (53, 54). The rate of adenosine removal can be reduced by hypoxia-dependent inhibition of adenosine kinase, resulting in further accumulation of adenosine (55, 56).

Inflammation consequently induces focal hypoxia in the inflamed tissue. Since inflammatory tissue damage involves non-specific injury to vasculature, disruption of blood circulation in the tissue causes deprivation of nutrients and oxygen downstream, and the resulting tissue hypoxia may increase extracellular adenosine production (57, 58). In addition, cellular damage, another consequence of proinflammatory activities, can result in the release of a large amount of adenine nucleotides, such as ATP, ADP, AMP, and adenosine, into the extracellular space. A combination of adenine nucleotide release and CD39/CD73 upregulation induces a robust accumulation of adenosine. Indeed, inflammation has been found to accompany an increase in extracellular adenosine levels (59–61). This hypoxia-induced metabolic switch favoring adenosine accumulation changes the course of inflammation. When inflammatory damage is excessive, the tissue will face an imminent danger, the loss of function. Adenosine produced from the hypoxic and damaged tissue can stop proinflammatory activities and, instead, facilitate anti-inflammatory activities (58). Moreover, adenosine promotes angiogenesis, which is an ultimate solution to tissue hypoxia, and tissue remodeling (62, 63). Therefore, adenosine upregulation in the hypoxic tissue can serve as a negative feedback mechanism of inflammation, promoting a switch from propagation of proinflammatory response to resolution of inflammation.

An important factor in keeping inflammation under control is the induction of a metabolic switch favoring adenosine accumulation. Just as A2AR deficiency caused exaggeration of inflammation, CD39- or CD73-deficient mice provided further evidence for the importance of endogenous adenosine in controlling inflammation (64–69). Although the release of intracellular adenosine from damaged cells can also increase extracellular adenosine levels, exaggerated inflammation in the absence of CD39 or CD73 indicates the pathophysiological significance of the metabolic adenosine production at the outer membrane. The importance of CD73 enzymatic activity was confirmed using an inhibitor of nucleotidase activity. It is also interesting that Treg cells express both CD39 and CD73, and the produced adenosine serves as an immunoregulatory mechanism of Treg cells (70–72).

Such biological significance of ectonucleotidases implied the role in the suppression of antitumor immunity. Interestingly, CD73 expression has been observed in mouse tumor cell lines (73–75) and tumor cells from various cancer patients (76–83). Those tumor cells are likely to generate adenosine by themselves for protection from immune cells. Inhibition of CD73 can reduce tumor growth (75), and indeed the suppression of tumor growth was found to be immune response dependent (84). Neutralization of CD73 by antibodies reduced tumor growth and metastasis from the primary tumor (84, 85). The reduction in tumor growth was not observed in immunocompromised mice, suggesting an involvement of antitumor immunity. Since anti-CD73 antibody did not block tumor growth in A2AR-deficient mice (84), it is likely that adenosine produced from CD73-expressing tumor cells suppresses antitumor immune response in an A2AR-dependent fashion. Supporting this speculation, knockdown of CD73 in tumor cells reduced their growth *in vivo* and increased their susceptibility to antitumor immune cells (84, 86). Tumor growth is also retarded in CD73-deficient mice, suggesting that adenosine production from non-tumor host cells also contributes to the establishment of the immunosuppressive environment (67, 68).

CD39, in conjunction with CD73, can promote adenosine accumulation and thereby inhibit immune activities in tumors. Deletion of CD39 from vasculature or bone marrow-derived cells as well as administration of a CD39 inhibitor blocked tumor growth in a model of hepatic metastasis (87). In human cancer, CD39 expression was observed in tumor cells, tumor stroma, and infiltrated lymphocytes (88). Inhibition of CD39 on tumor cells can relieve coexisting immune effector cells from immunosuppression (88, 89). Taken together, tumors often upregulate the extracellular adenosine-producing mechanism. Therefore, CD39 and CD73 are potential targets to block adenosine-dependent immunosuppression in tumors.

When mutated cells arise, immune cells may be able to recognize newly developed nascent tumor cells as abnormal and eliminate them (90). This immunosurveillance is considered to prevent cancer formation as shown by increased cancer incidence in immunocompromised mice that are lacking IFN-γ or perforin (91). Although most immune cells can exert a tumor-eliminating response, emergence of immune cells that produce anti-inflammatory cytokines or have immunoregulatory functions will change the microenvironment into tumor-permissive one. Since adenosine suppresses cellular immunity and promotes the switch to a tolerant immune profile, an abundance of extracellular adenosine may be detrimental to the prevention of cancer development. Consistent with this view, double transgenic mice expressing human papilloma virus type 16 E7 oncogene (E7) and A2AR were prone to spontaneous cancer development compared to E7-single transgenic mice. Thyroid specific coexpression of E7 and A2AR resulted in rapid development of thyroid carcinoma and lung metastasis of malignant thyroid cancer cells (92). Conversely, A2AR-deficient mice showed a reduced cancer incidence in 3-methylcholanthrene-induced chemical carcinogenesis (93). CD73-deficient mice were also resistant to 3-methylcholanthrene-induced carcinogenesis (68). CD73 deficiency is preventive to spontaneous prostate cancer development in TRAMP-transgenic mice (68). These studies suggest the significance of the adenosine system in cancer development, although the mechanism of cancer prevention needs further investigation.

## Non-Immune Targets of Adenosine in Cancer

As discussed above, immunosuppressive effects of extracellular adenosine provide tumors a protection from antitumor immunity. However, immune cells are not the only targets of the pro-tumor effect of adenosine. Some tumor cells have been found to express adenosine receptors, and adenosine may support tumor progression directly. In tumor cell culture *in vitro*, the addition of adenosine to the culture enhanced tumor cell proliferation. Cotreatment with antagonists suggested that the effect was mediated by A2AR or other adenosine receptors (94, 95). Adenosine can also prevent apoptosis of tumor cells, and A2AR antagonist reversed this effect (95). As discussed above, some tumor cells express CD73 and are able to increase extracellular adenosine levels. Tumor cell-derived adenosine not only protects the tumor from immune cells but also autonomously enhances tumor growth. Downregulation of CD73 in siRNA-transduced tumor cells reduced cell proliferation *in vitro* as well as tumor growth *in vivo* (75, 96). APCP, an inhibitor of enzymatic activity of CD73, also inhibited CD73-expressing tumor cell proliferation and increased apoptosis (94, 96). In contrast, CD73 overexpression increased tumor cell proliferation and viability (96).

CD73 expression on human cancer cells may induce resistance to chemotherapy. In human breast cancer cells, higher CD73 expression was associated with lower therapeutic response to anthracyclines (79). CD73 inhibitor sensitized the tumors to doxorubicin. In chronic lymphocytic leukemia, cancer cells constitutively express A2AR, and autonomous adenosine production with their own CD39/CD73 can result in A2AR-dependent prevention of drug-induced apoptosis (78). The inhibition of CD73 activity in glioblastoma cells decreased tumor cell viability to vincristine by downregulating multidrug transporter protein 1 (MRP1) (80).

Moreover, adenosine and CD73 expression may play a role in metastasis of cancer cells. CD73 downregulation by antibody or siRNA reduced adhesion, invasion, and migration of CD73-expressing tumor cells (75, 84). Conversely, CD73 overexpression increased adhesion and migration (97). When tumor cells were pretreated with anti-CD73 mAb before injection into mice, their extravasation and metastasis *in vivo* were decreased compared to control tumor cells (85). Addition of adenosine to the tumor cell culture increases tumor cell adhesion and migration (97, 98). Since adenosine does not increase tumor cell migration in the absence of A2AR (98), the adenosine-A2AR system may promote tumor metastasis by, in addition to its suppressive effect on antitumor immune response (29, 84, 99), directly activating tumor cell adhesion and migration. Although the pro-metastatic effect of CD73 may be mediated by adenosine production through its enzymatic activity, the non-enzymatic role of CD73 is also suggested in tumor cell migration (85).

## Tissue Hypoxia and Immunosuppression in Tumors

Thus, adenosine plays important roles in the establishment of an immunosuppressive tumor microenvironment and cancer progression. Adenosine is present in tumors at much higher levels than in normal tissues (18, 19). Tissue hypoxia seems to be crucial to the increase in intratumoral adenosine levels because reversal of tissue hypoxia by exposing tumor-bearing mice to hyperoxic atmosphere (60% oxygen) largely reduced tumor adenosine levels (100). Tumors are found to be hypoxic (101, 102) probably because of high oxygen demand by proliferating cells and insufficient oxygen supply due to sluggish blood flow in the disorganized tumor vasculature. Tissues under hypoxic stress shift adenosine metabolism toward its accumulation in the extracellular space. On one hand, hypoxia induces CD39 and CD73 and increases catabolic adenosine production (53, 103). On the other hand, hypoxia downregulates adenosine kinase and thereby inhibits conversion of adenosine (55, 56).

Corresponding to the increase in adenosine levels, hypoxia is known to be immunosuppressive. When cultured at various degrees of hypoxic atmosphere (1–5% oxygen), T cells undergo an impaired T cell receptor-mediated activation process (104) and reduce proliferation, cytokine production, and cytotoxicity (16, 105–108). T cell stimulation in mice breathing hypoxic atmosphere (10% oxygen) confirmed the impairment of T cell activation *in vivo* (16, 109). Consistent with the effects on T cells, hypoxia impairs the antigen-presenting function of dendritic cells and macrophages. Exposure of APCs to hypoxia downregulated both antigen uptake (110) and expression of MHC and costimulatory molecules (111, 112), resulting in the inability to achieve full activation of T cells. Immunosuppressive effects of hypoxia further include inhibition of NK cell activity (113) and induction of immunosuppressive activity of MDSCs (114). Although hypoxia conducts various functional changes through adenosine receptor-dependent mechanisms (115–117), an ­unidentified adenosine receptor-independent mechanism may also contribute to T cell suppression under hypoxia (16). While T cell proliferating activity is relatively resistant to A2AR-mediated T cell suppression, hypoxia strongly reduces T cell proliferation in an A2AR-independent manner (16, 31).

Nonetheless, tissue hypoxia, at least in part, contributes to immunosuppression in the tumor microenvironment. Many anti-inflammatory molecules are present in tumors; hypoxia may be responsible for the upregulation of some, if not all, of them. Reversal of hypoxia by whole body exposure to hyperoxic atmosphere (60% oxygen) reduced intratumoral expression of TGF-β, CTLA-4, COX-2, and FoxP3 (100, 118). In the same study, levels of CD39, CD73, A2AR, and A2BR in tumors were also reduced in response to hyperoxia, suggesting oxygen-dependent regulation of adenosine signaling pathway. Consistent with these changes indicating a switch to a more immuno-permissive tumor microenvironment, hyperoxia treatment inhibited tumor progression with an enhanced cellular immune response.

When deprived of oxygen, cells urgently need to prepare for the inevitable loss of oxidative energy production. To adapt to this dangerously stressful condition, cells quickly promote anaerobic energy production by glycolysis. A crucial molecule conducting this metabolic switch is HIF-1α, a transcriptional factor regulating glycolytic enzymes together with angiogenesis and erythropoiesis (119, 120). Tumor cells are known to utilize glycolysis even under aerobic conditions and satisfy their high energy demand with this all-out energy production. Therefore, it seems reasonable that increased HIF-1α levels in human cancers are positively correlated with the increased risk of mortality (121). While HIF-1α expression in tumors may indicate the presence of potentially immunosuppressive tissue hypoxia, HIF-1α may actively regulate immunosuppressive mechanisms. HIF-1α can mediate CD73 induction by hypoxia and promote adenosine-dependent immunosuppression (53). HIF-1α may be inhibitory to T cell activation as indicated by attenuated T cell receptor signaling (104). Indeed, T cells lacking HIF-1α showed stronger effector functions (122, 123) and caused severe inflammation (124). Contrary to the T cell suppressive role of HIF-1α, however, deletion of HIF-1α in myeloid cells diminished inflammation (125, 126). Bactericidal activities of macrophages and neutrophils are impaired when they lack HIF-1α (127). Interestingly, HIF-1α also positively regulates activity of MDSC, supporting their immunosuppressive activity, PD-L1 expression and conversion into tumor-associated macrophages (114, 128). These studies suggest that myeloid cells need HIF-1α for their activities, while HIF-1α may be inhibitory to T cell effector functions.

HIF-1α is also vital in controlling Treg cells, but its role is complex. Initially, hypoxia was reported to upregulate FoxP3 in mouse and human T cells (129). The induction of Treg cells in hypoxic conditions was confirmed (130) and extended in the demonstration of decreased immunoregulatory activity of HIF-1α-deficient Treg cells (131). Contrary to the positive role of HIF-1α in Treg cell induction, functional differentiation of HIF-1α-deficient CD4^+^ T cells demonstrated an increase in FoxP3^+^ Treg cells and a reciprocal decrease of Th17 cells (132, 133). The preferential differentiation into Treg cells in the absence of HIF-1 α was consistent with the reduced magnitude of inflammation in these animals. Inflammation was rather enhanced when Treg cells overexpress HIF-1α due to the lack of von Hippel–Lindau tumor suppressor (VHL), which degrades HIF-1α protein in normoxic conditions (134). Such a proinflammatory phenotype of these mice corresponded well with the reduction of FoxP3 and immunoregulatory activity. Similar changes were observed in Deltex1-deficient mice in which the lack of HIF-1α degradation led to its accumulation (135). In these studies, abundance of HIF-1α is considered to promote FoxP3 degradation leading to downregulation of Treg cells (133, 135). Nonetheless, even if roles of hypoxia and HIF-1α on Treg cell induction and immunregulatory activity are not clear, tumors contain a number of Treg cells. Another possibility for the increase of intratumoral Treg cell population is the active recruitment of Treg cells from the outside of tumors. Indeed, hypoxia triggers induction of a chemokine, CCL28, which attracts Treg cells into tumor tissues (136).

## Targeting the Hypoxia–Adenosine Pathway for Immune Checkpoint Therapy

For immune cells, the battle against tumors is a war that has to be fought in the enemy’s home ground. Although hypoxic tissue is a harsh environment for every cell, it may be a more challenging condition for immune cells than for tumor cells, which are ready to utilize various kinds of energy sources very aggressively. Since tumor cells drain energy sources as much as possible, it is hard for immune cells to keep on fighting in this scorched battleground. In addition, the inside of tumor tissue is a very hostile environment filled with a number of traps that will try to discourage the antitumor activity of immune cells. Many of such immunosuppressive activities are the use (abuse) of physiological immunoregulatory mechanisms that were originally intended to save vital organs from substantial inflammatory damage. Deceived by this false order, immune cells stop fighting tumor cells. As a result, immune cells, even highly activated immune effector cells that can recognize tumor-associated antigens, struggle mightily in the tumor. Hypoxia-inducible extracellular adenosine represents the physiological immunoregulatory molecules that are used to fortify tumors against immune system.

After adenosine-dependent tumor protection was first reported in 2006, extensive research on this pathway has revealed its significance in cancer immunology and cancer biology. Hypoxia and adenosine not only directly inhibits immune cell functions but also triggers induction of various anti-inflammatory molecules as well as emergence of immunoregulatory cells such as Treg cells, MDSC, and M2-type macrophages. The latter may contribute to establishing a long-lasting immunosuppressive environment in tumors. The pro-tumor effect of adenosine is not limited to immunosuppression, and it involves non-immunological pro-tumor effects, which promote tumor cell proliferation, tumor cell survival, metastasis, and angiogenesis. Clinical data support the pro-tumor effects of the hypoxia–adenosine pathway. Many cancer cells express CD73, and CD73 expression is associated with poor prognosis of various cancers such as breast (79), ovarian (77, 83), prostate (81, 82), brain cancers (80), and leukemia (76, 78). Resistance to chemotherapy has been reported in CD73-expressing cancers (78–80). An association with increased mortality has also been observed in cancers with poorly oxygenated areas (102, 137, 138) and in cancers expressing HIF-1α at high levels (139, 140). Corresponding to the poor prognosis, hypoxic cancers are found to be refractory to radiotherapy and chemotherapy.

**Figure 1:**
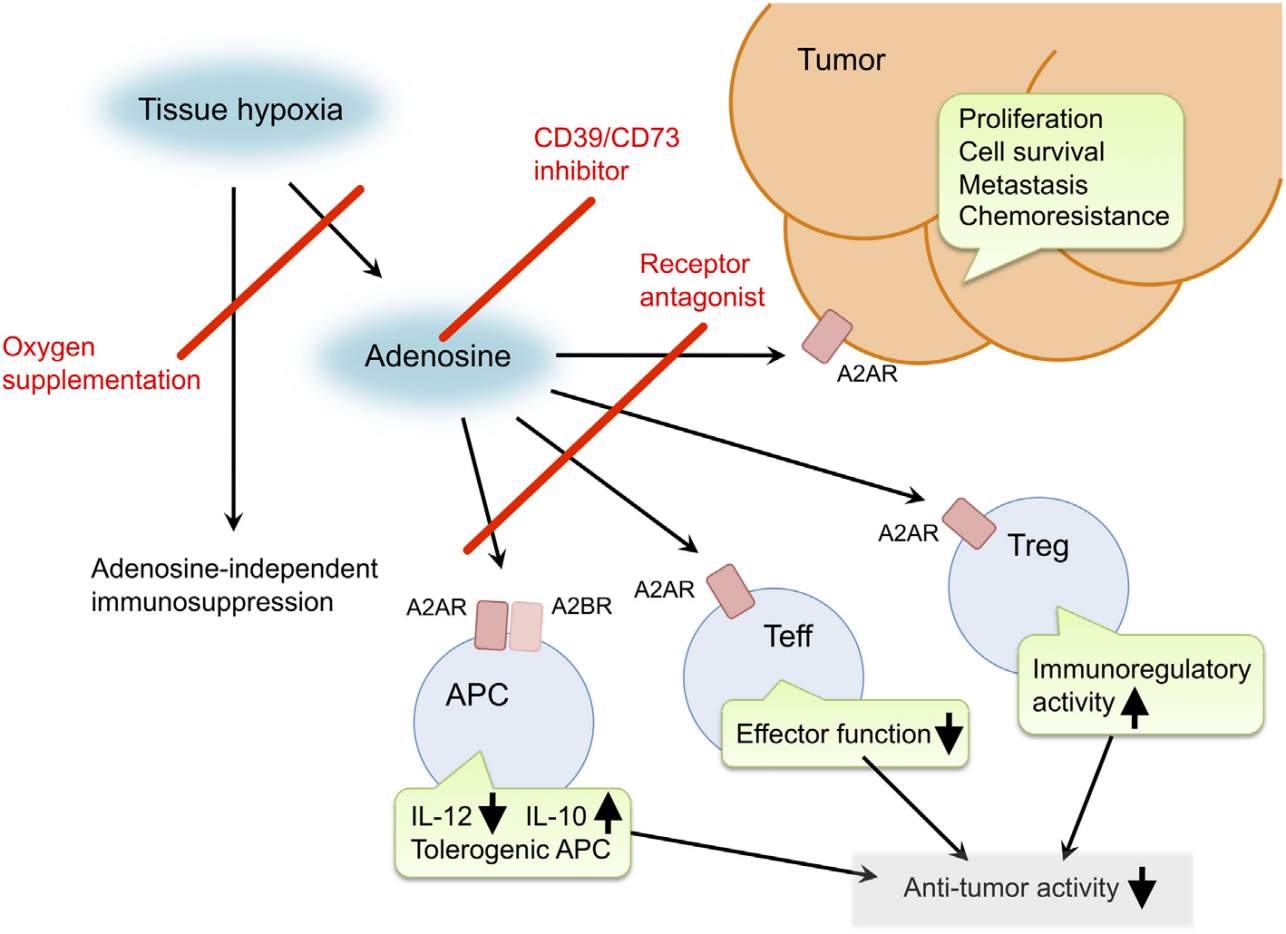
**Pro-cancer activities of extracellular adenosine and therapeutic targets for improvement of cancer immunotherapy**. Tissue hypoxia in tumors increases extracellular adenosine production through induction of CD39 and CD73. Produced adenosine transmits immunosuppressive signals through adenosine receptors on various immune cells. A2AR stimulation in effector T cells (Teff) blocks T cell receptor signaling and impairs effector functions including IFN-γ production and cytotoxicity. In antigen-presenting cells (APC), signals through A2AR and A2BR reduce Th1-type cytokine milieu and induce tolerogenic APC. Interaction of Teff with these APC will impair activation of cellular immune response against cancer cells. Adenosine enhances immunoregulatory activity of regulatory T cells (Treg). The qualitative and quantitative increase of Treg results in stronger inactivation of antitumor immune response. In addition, adenosine can promote proliferation, survival and metastatic activity of cancer cells. Suppression of adenosine pathway will be able to weaken the intensity of immunosuppression in tumor microenvironment and direct effect on cancer cells. Potential target molecules are adenosine receptors (A2AR and A2BR) and adenosine-producing enzymes (CD39 and CD73). Oxygen supplementation can also decrease pro-cancer effects of adenosine as well as adenosine-independent immunosuppression by hypoxia.

Inactivation of the hypoxia–adenosine pathway may be able to relieve antitumor immune effector cells from immunosuppressive signaling, convert an immunosuppressive tumor microenvironment to a more immuno-permissive one, and reduce metastatic activity and resistance to chemotherapy and radiotherapy (Figure 1). The pathway can be targeted in different steps. Antagonists of adenosine receptors block adenosine signaling and improve tumor regression (19, 21, 41). Reduction of extracellular adenosine levels by CD73 inhibitor also improves tumor regression (141, 142). Improvement of tissue oxygenation status reduces adenosine levels and enhances immune cell activities as observed in mice breathing hyperoxic atmosphere (100, 118). When combined with therapeutic approaches that increase the number of antitumor cells, the anti-hypoxia–adenosine strategy may synergistically enhance efficacy by preventing inactivation of effector cells. Preclinical studies have shown further retardation of tumor growth and reduction of metastatic tumor nodules in combined treatments with adoptive immunotherapy (19, 86) or cancer vaccines (143). Interestingly, anti-hypoxia–adenosine therapy cooperates well with other immune checkpoint inhibitors such as ani-CTLA-4 and anti-PD-1 mAbs (29, 34, 47, 144). The complexity of regulating antitumor immunity may limit success in treatments targeting a single mechanism. However, combined treatment with the anti-hypoxia–adenosine strategy may improve the clinical response to other immunotherapy as well as chemotherapy and radiotherapy.

## Author Contributions

AO conceived, wrote, and edited the manuscript.

## Conflict of Interest Statement

The author declares that the research was conducted in the absence of any commercial or financial relationships that could be construed as a potential conflict of interest.
